# Tetra­kis(8-quinolinolato-κ^2^ *N*,*O*)hafnium(IV) dimethyl­formamide solvate monohydrate

**DOI:** 10.1107/S1600536810014960

**Published:** 2010-04-30

**Authors:** Johannes A. Viljoen, Hendrik G. Visser, Andreas Roodt

**Affiliations:** a Department of Chemistry, University of the Free State, PO Box 339, Bloemfontein 9300, South Africa

## Abstract

In the title compound, [Hf(C_9_H_6_NO)]·C_3_H_7_NO·H_2_O, the hafnium(IV) atom is coordinated by four 8-quinolinolate (Ox) ligands, forming a slightly distorted square-anti­prismatic coordination polyhedron. The crystal packing is controlled by O—H⋯O and C—H⋯O hydrogen-bonding inter­actions and π–π inter­actions between quinoline ligands of neighbouring mol­ecules. The inter­planar distances vary between 3.150 (1) and 3.251 (2) Å, while centroid–centroid distances vary from 3.589 (1) to 4.1531 (1) Å.

## Related literature

For other solvates of the title compound crystallizing in *P* 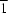 and *Fddd*, see: Viljoen *et al.* (2009*a* ▶) and Lewis & Fay (1974 ▶), respectively. For hafnium and zirconium *β*-diketonato complexes, see: Viljoen *et al.* (2008 ▶, 2009*b* ▶); Demakopoulos *et al.* (1995 ▶); Zherikova *et al.* (2005 ▶, 2006 ▶, 2008 ▶); Steyn *et al.* (2008 ▶); Calderazzo *et al.* (1998 ▶). For acetyl­acetone in separation chemistry, see: Van Aswegen *et al.* (1991 ▶); Steyn *et al.* (1992 ▶, 1997 ▶); Otto *et al.* (1998 ▶); Roodt & Steyn (2000 ▶); Brink *et al.* (2010 ▶). 
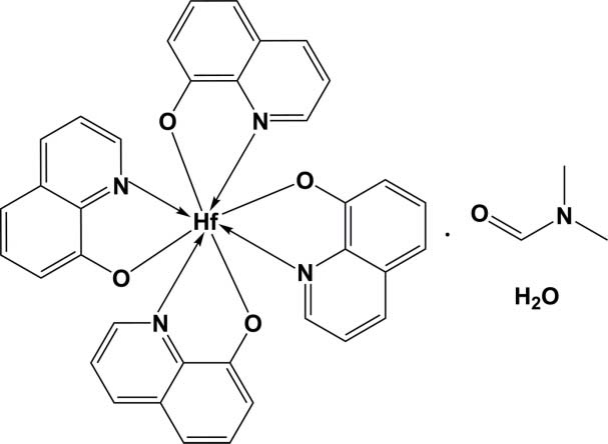


## Experimental

### 

#### Crystal data

[Hf(C_9_H_6_NO)]·C_3_H_7_NO·H_2_O
*M* *_r_* = 846.19
Triclinic, 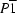

*a* = 11.360 (5) Å
*b* = 12.245 (4) Å
*c* = 12.504 (5) Å
α = 91.817 (4)°
β = 103.333 (5)°
γ = 99.190 (5)°
*V* = 1666.5 (11) Å^3^
*Z* = 2
Mo *K*α radiation
μ = 3.19 mm^−1^
*T* = 100 K
0.44 × 0.36 × 0.33 mm

#### Data collection

Bruker X8 APEXII 4K Kappa CCD diffractometer
Absorption correction: multi-scan (*SADABS*; Bruker, 2004 ▶) *T*_min_ = 0.262, *T*_max_ = 0.349
28187 measured reflections
7242 independent reflections
6906 reflections with *I* > 2σ(*I*)
*R*_int_ = 0.037

#### Refinement

*R*[*F* ^2^ > 2σ(*F* ^2^)] = 0.022
*wR*(*F* ^2^) = 0.053
*S* = 1.08
7242 reflections
471 parameters
3 restraints
H atoms treated by a mixture of independent and constrained refinement
Δρ_max_ = 1.35 e Å^−3^
Δρ_min_ = −0.98 e Å^−3^

### 

Data collection: *APEX2* (Bruker, 2005 ▶); cell refinement: *SAINT-Plus* (Bruker, 2004 ▶); data reduction: *SAINT-Plus*; program(s) used to solve structure: *SIR92* (Altomare *et al.*, 1999 ▶); program(s) used to refine structure: *SHELXL97* (Sheldrick, 2008 ▶); molecular graphics: *DIAMOND* (Brandenburg & Putz, 2005 ▶); software used to prepare material for publication: *WinGX* (Farrugia, 1999 ▶).

## Supplementary Material

Crystal structure: contains datablocks I, global. DOI: 10.1107/S1600536810014960/rk2202sup1.cif

Structure factors: contains datablocks I. DOI: 10.1107/S1600536810014960/rk2202Isup2.hkl

Additional supplementary materials:  crystallographic information; 3D view; checkCIF report

## Figures and Tables

**Table 1:** Hydrogen-bond geometry (Å, °)

*D*—H⋯*A*	*D*—H	H⋯*A*	*D*⋯*A*	*D*—H⋯*A*
O01—H02⋯O2	0.89 (2)	1.99 (2)	2.867 (3)	171 (3)
O01—H01⋯O001	0.95 (2)	1.83 (2)	2.757 (4)	164 (4)
C31—H31⋯O001	0.95	2.51	3.418 (4)	160
C004—H004⋯O01^i^	0.95	2.41	3.331 (5)	164

Symmetry code: (i) 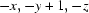.

**Fig. 1.**
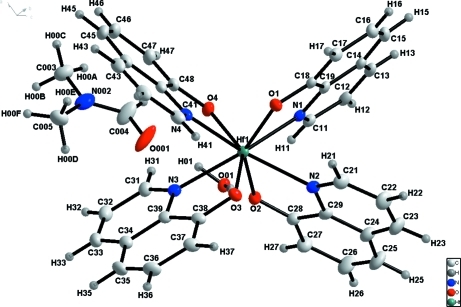
Representation of the title compound, showing the atoms numbering scheme. Displacement ellipsoids are drawn at the 50% probability level. H atoms are presented as a small spheres of arbitrary radius.

**Fig. 2.**
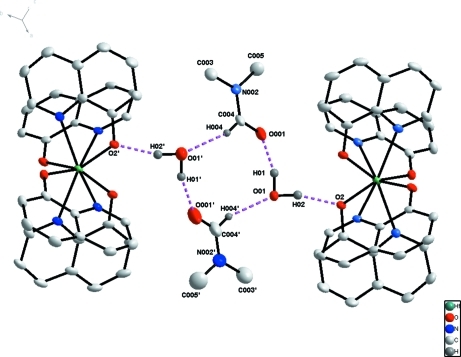
Graphical illustration of Hf(Ox)4 indicating C–H···O and O–H···O hydrogen bonding interaction is observed between the solvent molecules and one of the oxygen atoms from a neighbouring metallic molecular group (displacement ellipsoids are drawn at the 50% probability level). Symmetry codes: (i) -x, 1-y, -z.

**Fig. 3.**
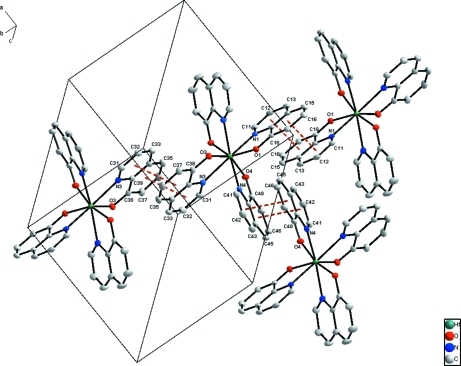
Graphical illustration of π–π interaction and stacking between different quinoline ligands of neighbouring molecules to form a three dimensional network (displacement ellipsoids are drawn at the 50% probability level). Hydrogen atoms omitted for clarity.

## supplementary crystallographic information

### Comment

Acetylacetone and bidentate ligand analogues find applications in homogenous catalysis as model precursors. However, it is also utilized in the extraction and separation industry (Van Aswegen *et al.*, (1991); Steyn *et al.*, (1992, 1997); Otto *et al.*, (1998); Roodt & Steyn, (2000); Brink *et al.*, (2010)). This study forms part of an ongoing research project that investigates the formation of Hf(IV) and Zr(IV) complexes with various bidentate ligands with possible applications in the mentioned industries (Zherikova *et al.*, (2005, 2006, 2008); Steyn *et al.*, (2008); Viljoen *et al.*, (2008, 2009a,b); Demakopoulos *et al.*, (1995); Lewis & Fay (1974) and Calderazzo *et al.* (1998).

Orange cubic-like crystals of the title complex crystallize (Fig. 1) with both an aqua and a dimethylformamide solvent molecule in the asymmetric unit. The Hf(IV) atom is eight coordinated and surrounded by four *N,O*-bidentate (Ox) ligands to give a slightly distorted square antiprismatic coordination geometry. The Hf–O and Hf–N bond lengths vary from 2.080 (2)Å to 2.115 (2)Å and 2.389 (2)Å to 2.411 (2) Å, respectivily, and the O–Hf–N bite angles vary from 70.7 (1)° to 71.2 (1)°.

Strong C–H···O and O–H···O hydrogen bonding interactions are observed between the solvent molecules and one of the oxygen atoms of a neighbouring complex molecule (Table 1 & Fig. 2). The dihedral angle between the two phenyl rings of the Ox ligands are all less than 2° (rings 1, 2, 3 & 4 being 0.514 (12)°, 0.595 (9)°, 1.873 (9)° and 1.566 (10)°, respectively), indicating little or negligible distortion due to coordination or packing. The molecular units of title complex are packed in a head-to-head fashion along the *ac* plane and are connected by π–π interactions between different Ox ligands of neighbouring molecules to produce a three dimensional network, with interplaner distances varying between 3.150 (1)Å and 3.251 (2)Å and centroid-to-centroid distances from 3.589 (1)Å to 4.1531 (1)Å (Fig. 3).

### Experimental

Chemicals were purchased from Sigma-Aldrich and used as received. HfCl_4_ (603 mg, 1.9 mmol) was dissolved in a minimal amount of *DMF*. While stirring this solution at room temperature, another solution of C_9_H_7_ON (1.07 g, 7.4 mmol) was dissolved in a minimal amount of *DMF* and slowly added to the HfCl_4_ solution, resulting in the formation of a bright yellow solution. The solution was left to stand for *ca*. a week for crystals to form. (Yield: 1.32 g, 92%). Spectroscopy data: ^1^H NMR (Benzene-*d*_6_): *δ* = 6.73 (d, 1H, *J* = 6 Hz), 7.31 (dd, 2H, *J* = 7.8 Hz, 6 Hz), 7.40 (t, 2H, *J* = 7.8 Hz), 8.11 (d, 1H, *J* = 7.2 Hz); IR (ATR): *ν*(CO) 1666 cm^-1^.

### Refinement

The aromatic, methine, and methyl H atoms were placed in geometrically idealized positions (C–H = 0.95Å or 0.98 Å) and constrained to ride on their parent atoms with *U*_iso_(H) = 1.2*U*_eq_(C) for aromatic and methine, and *U*_iso_(H) = 1.5*U*_eq_(C) for methyl protons. Torsion angles for methyl protons were refined from electron density. The hydrogen atoms of the solvent water molecule were located on the Fourier difference map and refined isotropically. The highest residual electron density was located 2.23Å from H41 and was essentially meaningless.

### Crystal data

**Table:**

[Hf(C_9_H_6_NO)]·C_3_H_7_NO·H_2_O	*Z* = 2
*M**_r_* = 846.19	*F*(000) = 844
Triclinic, *P*1	*D*_x_ = 1.686 Mg m^−^^3^
Hall symbol: -P 1	Mo *K*α radiation, λ = 0.71069 Å
*a* = 11.360 (5) Å	Cell parameters from 9880 reflections
*b* = 12.245 (4) Å	θ = 2.2–28.3°
*c* = 12.504 (5) Å	µ = 3.19 mm^−^^1^
α = 91.817 (4)°	*T* = 100 K
β = 103.333 (5)°	Cuboid, orange
γ = 99.190 (5)°	0.44 × 0.36 × 0.33 mm
*V* = 1666.5 (11) Å^3^	

### Data collection

**Table:**

Bruker X8 APEXII 4K Kappa CCD diffractometer	7242 independent reflections
Radiation source: fine-focus sealed tube	6906 reflections with *I* > 2σ(*I*)
graphite	*R*_int_ = 0.037
ω and φ scans	θ_max_ = 27°, θ_min_ = 1.9°
Absorption correction: multi-scan (*SADABS*; Bruker, 2004)	*h* = −14→14
*T*_min_ = 0.262, *T*_max_ = 0.349	*k* = −15→15
28187 measured reflections	*l* = −15→15

### Refinement

**Table:**

Refinement on *F*^2^	Primary atom site location: structure-invariant direct methods
Least-squares matrix: full	Secondary atom site location: difference Fourier map
*R*[*F*^2^ > 2σ(*F*^2^)] = 0.022	Hydrogen site location: inferred from neighbouring sites
*wR*(*F*^2^) = 0.053	H atoms treated by a mixture of independent and constrained refinement
*S* = 1.08	*w* = 1/[σ^2^(*F*_o_^2^) + (0.0127*P*)^2^ + 2.7005*P*] where *P* = (*F*_o_^2^ + 2*F*_c_^2^)/3
7242 reflections	(Δ/σ)_max_ = 0.004
471 parameters	Δρ_max_ = 1.35 e Å^−^^3^
3 restraints	Δρ_min_ = −0.98 e Å^−^^3^

### Special details

**Table:**

Geometry. All s.u.'s (except the s.u. in the dihedral angle between two l.s. planes) are estimated using the full covariance matrix. The cell s.u.'s are taken into account individually in the estimation of s.u.'s in distances, angles and torsion angles; correlations between s.u.'s in cell parameters are only used when they are defined by crystal symmetry. An approximate (isotropic) treatment of cell s.u.'s is used for estimating s.u.'s involving l.s. planes.
Refinement. Refinement of *F*^2^ against ALL reflections. The weighted *R*-factor w*R* and goodness of fit *S* are based on *F*^2^, conventional *R*-factors *R* are based on *F*, with *F* set to zero for negative *F*^2^. The threshold expression of *F*^2^ > 2σ(*F*^2^) is used only for calculating *R*-factors(gt) etc. and is not relevant to the choice of reflections for refinement. *R*-factors based on *F*^2^ are statistically about twice as large as those based on *F*, and *R*-factors based on ALL data will be even larger.

### Fractional atomic coordinates and isotropic or equivalent isotropic displacement parameters (Å^2^)

**Table:**

	*x*	*y*	*z*	*U*_iso_*/*U*_eq_	
C11	0.1792 (2)	0.1576 (2)	0.0254 (2)	0.0168 (5)	
H11	0.1959	0.2364	0.0321	0.02*	
C12	0.1362 (2)	0.1030 (2)	−0.0806 (2)	0.0190 (6)	
H12	0.1268	0.1446	−0.144	0.023*	
C003	−0.2444 (4)	0.5958 (4)	0.1882 (4)	0.0517 (10)	
H00A	−0.2626	0.5958	0.1077	0.078*	
H00B	−0.2424	0.6705	0.22	0.078*	
H00C	−0.3083	0.5436	0.2099	0.078*	
C13	0.1081 (2)	−0.0100 (2)	−0.0917 (2)	0.0200 (6)	
H13	0.0773	−0.0474	−0.1629	0.024*	
C004	−0.0657 (4)	0.5254 (4)	0.1614 (4)	0.0596 (13)	
H004	−0.1022	0.519	0.0845	0.071*	
C14	0.1252 (2)	−0.0713 (2)	0.0029 (2)	0.0174 (5)	
C15	0.0986 (3)	−0.1884 (2)	0.0019 (2)	0.0224 (6)	
H15	0.0668	−0.2318	−0.066	0.027*	
C005	−0.0741 (4)	0.5701 (4)	0.3460 (4)	0.0541 (11)	
H00D	0.0134	0.6017	0.3612	0.081*	
H00E	−0.084	0.4958	0.3734	0.081*	
H00F	−0.1164	0.6177	0.3831	0.081*	
C16	0.1189 (3)	−0.2386 (2)	0.0991 (2)	0.0226 (6)	
H16	0.1026	−0.3173	0.0977	0.027*	
C17	0.1634 (3)	−0.1762 (2)	0.2018 (2)	0.0185 (6)	
H17	0.1763	−0.2135	0.2679	0.022*	
C18	0.1883 (2)	−0.0617 (2)	0.2071 (2)	0.0153 (5)	
C19	0.1703 (2)	−0.0094 (2)	0.1058 (2)	0.0156 (5)	
C21	0.4753 (3)	0.0155 (2)	0.2458 (2)	0.0218 (6)	
H21	0.4464	−0.033	0.2955	0.026*	
C22	0.5586 (3)	−0.0176 (3)	0.1892 (3)	0.0274 (7)	
H22	0.5848	−0.0872	0.2003	0.033*	
C23	0.6015 (3)	0.0514 (3)	0.1181 (3)	0.0295 (7)	
H23	0.6561	0.029	0.0777	0.035*	
C24	0.5653 (3)	0.1558 (3)	0.1042 (2)	0.0236 (6)	
C25	0.6040 (3)	0.2345 (3)	0.0332 (3)	0.0325 (7)	
H25	0.6604	0.2194	−0.0085	0.039*	
C26	0.5602 (3)	0.3317 (3)	0.0250 (3)	0.0315 (7)	
H26	0.5867	0.3838	−0.0229	0.038*	
C27	0.4761 (3)	0.3576 (2)	0.0858 (2)	0.0238 (6)	
H27	0.447	0.4262	0.078	0.029*	
C28	0.4361 (3)	0.2839 (2)	0.1560 (2)	0.0181 (5)	
C29	0.4814 (2)	0.1817 (2)	0.1647 (2)	0.0170 (5)	
C31	0.2530 (3)	0.4285 (2)	0.4055 (2)	0.0191 (6)	
H31	0.1904	0.4287	0.3404	0.023*	
C32	0.2814 (3)	0.5200 (2)	0.4831 (2)	0.0235 (6)	
H32	0.2385	0.5807	0.4699	0.028*	
C33	0.3708 (3)	0.5216 (2)	0.5774 (2)	0.0239 (6)	
H33	0.3904	0.5834	0.6301	0.029*	
C34	0.4339 (3)	0.4311 (2)	0.5966 (2)	0.0194 (6)	
C35	0.5283 (3)	0.4232 (3)	0.6904 (2)	0.0248 (6)	
H35	0.5517	0.4806	0.7479	0.03*	
C36	0.5858 (3)	0.3330 (3)	0.6985 (2)	0.0243 (6)	
H36	0.6484	0.3284	0.7625	0.029*	
C37	0.5549 (3)	0.2463 (2)	0.6142 (2)	0.0209 (6)	
H37	0.5979	0.1854	0.621	0.025*	
C38	0.4622 (3)	0.2505 (2)	0.5219 (2)	0.0175 (5)	
C39	0.4008 (2)	0.3433 (2)	0.5136 (2)	0.0165 (5)	
C41	0.2162 (3)	0.0956 (2)	0.5456 (2)	0.0206 (6)	
H41	0.2943	0.0731	0.5603	0.025*	
C42	0.1450 (3)	0.0799 (2)	0.6242 (2)	0.0243 (6)	
H42	0.1755	0.0477	0.6909	0.029*	
C43	0.0329 (3)	0.1109 (2)	0.6045 (2)	0.0258 (7)	
H43	−0.0152	0.1002	0.6574	0.031*	
C44	−0.0125 (3)	0.1594 (2)	0.5050 (2)	0.0218 (6)	
C45	−0.1292 (3)	0.1914 (3)	0.4740 (3)	0.0270 (7)	
H45	−0.1836	0.1824	0.5217	0.032*	
C46	−0.1630 (3)	0.2353 (3)	0.3746 (3)	0.0278 (7)	
H46	−0.2413	0.2569	0.3543	0.033*	
C47	−0.0846 (3)	0.2494 (3)	0.3008 (2)	0.0242 (6)	
H47	−0.1105	0.2815	0.233	0.029*	
C48	0.0284 (3)	0.2172 (2)	0.3266 (2)	0.0186 (6)	
C49	0.0651 (3)	0.1725 (2)	0.4312 (2)	0.0178 (6)	
N1	0.1973 (2)	0.10354 (19)	0.11572 (18)	0.0150 (4)	
N2	0.4355 (2)	0.11053 (19)	0.23296 (18)	0.0166 (5)	
N002	−0.1265 (3)	0.5625 (2)	0.2283 (2)	0.0347 (7)	
N3	0.3101 (2)	0.34199 (18)	0.41944 (18)	0.0164 (5)	
N4	0.1772 (2)	0.14071 (18)	0.45181 (18)	0.0167 (5)	
O01	0.2193 (2)	0.4529 (2)	0.0961 (2)	0.0365 (6)	
O1	0.22623 (17)	0.00277 (15)	0.29999 (14)	0.0167 (4)	
O2	0.35700 (17)	0.30047 (15)	0.21652 (15)	0.0168 (4)	
O3	0.42709 (17)	0.17339 (15)	0.43809 (15)	0.0165 (4)	
O4	0.10612 (17)	0.22174 (16)	0.26121 (15)	0.0183 (4)	
O001	0.0360 (4)	0.4985 (4)	0.1927 (3)	0.0962 (16)	
Hf1	0.279295 (10)	0.175184 (9)	0.304673 (8)	0.01335 (4)	
H01	0.156 (3)	0.456 (4)	0.134 (3)	0.064 (14)*	
H02	0.258 (3)	0.408 (3)	0.139 (3)	0.051 (12)*	

### Atomic displacement parameters (Å^2^)

**Table:**

	*U*^11^	*U*^22^	*U*^33^	*U*^12^	*U*^13^	*U*^23^
C11	0.0141 (13)	0.0219 (14)	0.0158 (13)	0.0036 (11)	0.0059 (10)	0.0026 (10)
C12	0.0137 (13)	0.0332 (16)	0.0117 (12)	0.0064 (12)	0.0044 (10)	0.0029 (11)
C003	0.039 (2)	0.063 (3)	0.054 (3)	0.006 (2)	0.0148 (19)	0.014 (2)
C13	0.0150 (13)	0.0314 (16)	0.0142 (13)	0.0044 (12)	0.0052 (11)	−0.0033 (11)
C004	0.064 (3)	0.084 (3)	0.053 (3)	0.040 (3)	0.035 (2)	0.044 (2)
C14	0.0120 (13)	0.0235 (14)	0.0181 (13)	0.0030 (11)	0.0068 (10)	−0.0029 (11)
C15	0.0194 (14)	0.0269 (15)	0.0211 (14)	0.0049 (12)	0.0059 (12)	−0.0080 (12)
C005	0.046 (2)	0.053 (3)	0.055 (3)	−0.001 (2)	0.003 (2)	−0.008 (2)
C16	0.0210 (15)	0.0206 (14)	0.0271 (15)	0.0046 (12)	0.0077 (12)	−0.0043 (12)
C17	0.0170 (14)	0.0190 (13)	0.0208 (14)	0.0055 (11)	0.0056 (11)	0.0019 (11)
C18	0.0105 (12)	0.0210 (13)	0.0153 (13)	0.0030 (10)	0.0052 (10)	−0.0009 (10)
C19	0.0118 (12)	0.0214 (14)	0.0155 (13)	0.0047 (10)	0.0057 (10)	−0.0003 (10)
C21	0.0197 (14)	0.0223 (14)	0.0237 (15)	0.0062 (12)	0.0035 (12)	0.0053 (11)
C22	0.0237 (16)	0.0292 (16)	0.0326 (17)	0.0144 (13)	0.0061 (13)	0.0054 (13)
C23	0.0209 (16)	0.0361 (18)	0.0368 (18)	0.0118 (14)	0.0130 (14)	0.0028 (14)
C24	0.0177 (14)	0.0298 (16)	0.0254 (15)	0.0053 (12)	0.0085 (12)	0.0033 (12)
C25	0.0298 (18)	0.0410 (19)	0.0353 (18)	0.0098 (15)	0.0220 (15)	0.0086 (15)
C26	0.0352 (18)	0.0341 (18)	0.0325 (17)	0.0056 (15)	0.0215 (15)	0.0121 (14)
C27	0.0284 (16)	0.0206 (14)	0.0256 (15)	0.0036 (12)	0.0125 (13)	0.0065 (12)
C28	0.0166 (14)	0.0195 (13)	0.0182 (13)	0.0013 (11)	0.0055 (11)	0.0006 (10)
C29	0.0118 (13)	0.0223 (14)	0.0157 (13)	0.0014 (11)	0.0017 (10)	0.0013 (10)
C31	0.0225 (15)	0.0202 (14)	0.0166 (13)	0.0048 (11)	0.0076 (11)	0.0036 (10)
C32	0.0275 (16)	0.0186 (14)	0.0275 (15)	0.0046 (12)	0.0120 (13)	0.0023 (12)
C33	0.0254 (16)	0.0222 (14)	0.0256 (15)	0.0002 (12)	0.0121 (13)	−0.0040 (12)
C34	0.0216 (14)	0.0198 (14)	0.0165 (13)	−0.0014 (11)	0.0074 (11)	0.0001 (10)
C35	0.0260 (16)	0.0267 (15)	0.0176 (14)	−0.0047 (12)	0.0042 (12)	−0.0048 (11)
C36	0.0214 (15)	0.0298 (16)	0.0167 (14)	−0.0015 (12)	−0.0019 (11)	0.0032 (12)
C37	0.0207 (14)	0.0206 (14)	0.0190 (14)	0.0011 (11)	0.0009 (11)	0.0056 (11)
C38	0.0185 (14)	0.0185 (13)	0.0136 (12)	−0.0029 (11)	0.0041 (11)	0.0027 (10)
C39	0.0174 (13)	0.0165 (13)	0.0159 (13)	−0.0004 (11)	0.0065 (11)	0.0032 (10)
C41	0.0269 (15)	0.0181 (13)	0.0147 (13)	−0.0023 (11)	0.0053 (11)	0.0010 (10)
C42	0.0353 (17)	0.0221 (14)	0.0144 (13)	−0.0045 (13)	0.0100 (12)	0.0012 (11)
C43	0.0343 (18)	0.0232 (15)	0.0203 (14)	−0.0067 (13)	0.0159 (13)	−0.0021 (11)
C44	0.0264 (16)	0.0199 (14)	0.0182 (14)	−0.0044 (12)	0.0101 (12)	−0.0062 (11)
C45	0.0235 (16)	0.0314 (17)	0.0278 (16)	−0.0012 (13)	0.0150 (13)	−0.0083 (13)
C46	0.0198 (15)	0.0352 (17)	0.0291 (16)	0.0041 (13)	0.0092 (13)	−0.0109 (13)
C47	0.0218 (15)	0.0308 (16)	0.0201 (14)	0.0050 (12)	0.0054 (12)	−0.0058 (12)
C48	0.0192 (14)	0.0210 (14)	0.0149 (13)	0.0001 (11)	0.0059 (11)	−0.0056 (10)
C49	0.0204 (14)	0.0158 (13)	0.0167 (13)	−0.0026 (11)	0.0079 (11)	−0.0044 (10)
N1	0.0119 (11)	0.0209 (12)	0.0130 (11)	0.0027 (9)	0.0045 (9)	0.0007 (9)
N2	0.0144 (11)	0.0199 (12)	0.0149 (11)	0.0026 (9)	0.0023 (9)	0.0020 (9)
N002	0.0285 (15)	0.0356 (16)	0.0429 (17)	0.0020 (12)	0.0160 (13)	0.0101 (13)
N3	0.0181 (12)	0.0182 (11)	0.0130 (11)	0.0002 (9)	0.0055 (9)	0.0028 (9)
N4	0.0211 (12)	0.0148 (11)	0.0132 (11)	−0.0023 (9)	0.0057 (9)	−0.0019 (8)
O01	0.0390 (14)	0.0417 (14)	0.0333 (13)	0.0178 (12)	0.0091 (11)	0.0111 (11)
O1	0.0196 (10)	0.0176 (9)	0.0118 (9)	−0.0007 (8)	0.0048 (8)	−0.0003 (7)
O2	0.0193 (10)	0.0171 (9)	0.0156 (9)	0.0026 (8)	0.0075 (8)	0.0022 (7)
O3	0.0189 (10)	0.0145 (9)	0.0149 (9)	0.0030 (7)	0.0014 (8)	0.0012 (7)
O4	0.0190 (10)	0.0244 (10)	0.0129 (9)	0.0049 (8)	0.0059 (8)	0.0012 (7)
O001	0.085 (3)	0.167 (4)	0.085 (3)	0.093 (3)	0.060 (2)	0.089 (3)
Hf1	0.01496 (6)	0.01537 (6)	0.00978 (6)	0.00116 (4)	0.00402 (4)	0.00116 (4)

### Geometric parameters (Å, °)

**Table:**

C11—N1	1.319 (3)	C31—N3	1.324 (4)
C11—C12	1.409 (4)	C31—C32	1.404 (4)
C11—H11	0.95	C31—H31	0.95
C12—C13	1.365 (4)	C32—C33	1.365 (4)
C12—H12	0.95	C32—H32	0.95
C003—N002	1.445 (5)	C33—C34	1.411 (4)
C003—H00A	0.98	C33—H33	0.95
C003—H00B	0.98	C34—C35	1.413 (4)
C003—H00C	0.98	C34—C39	1.412 (4)
C13—C14	1.414 (4)	C35—C36	1.365 (4)
C13—H13	0.95	C35—H35	0.95
C004—O001	1.232 (5)	C36—C37	1.415 (4)
C004—N002	1.314 (5)	C36—H36	0.95
C004—H004	0.95	C37—C38	1.380 (4)
C14—C19	1.415 (4)	C37—H37	0.95
C14—C15	1.416 (4)	C38—O3	1.332 (3)
C15—C16	1.369 (4)	C38—C39	1.420 (4)
C15—H15	0.95	C39—N3	1.372 (4)
C005—N002	1.449 (5)	C41—N4	1.326 (4)
C005—H00D	0.98	C41—C42	1.408 (4)
C005—H00E	0.98	C41—H41	0.95
C005—H00F	0.98	C42—C43	1.358 (5)
C16—C17	1.415 (4)	C42—H42	0.95
C16—H16	0.95	C43—C44	1.419 (4)
C17—C18	1.381 (4)	C43—H43	0.95
C17—H17	0.95	C44—C45	1.414 (4)
C18—O1	1.327 (3)	C44—C49	1.413 (4)
C18—C19	1.425 (4)	C45—C46	1.367 (5)
C19—N1	1.363 (4)	C45—H45	0.95
C21—N2	1.316 (4)	C46—C47	1.419 (4)
C21—C22	1.406 (4)	C46—H46	0.95
C21—H21	0.95	C47—C48	1.373 (4)
C22—C23	1.363 (4)	C47—H47	0.95
C22—H22	0.95	C48—O4	1.331 (3)
C23—C24	1.408 (4)	C48—C49	1.432 (4)
C23—H23	0.95	C49—N4	1.361 (4)
C24—C29	1.413 (4)	N1—Hf1	2.411 (2)
C24—C25	1.417 (4)	N2—Hf1	2.391 (2)
C25—C26	1.360 (5)	N3—Hf1	2.389 (2)
C25—H25	0.95	N4—Hf1	2.405 (2)
C26—C27	1.417 (4)	O01—H01	0.95 (2)
C26—H26	0.95	O01—H02	0.89 (2)
C27—C28	1.376 (4)	O1—Hf1	2.096 (2)
C27—H27	0.95	O2—Hf1	2.1145 (19)
C28—O2	1.336 (3)	O3—Hf1	2.0796 (19)
C28—C29	1.425 (4)	O4—Hf1	2.092 (2)
C29—N2	1.366 (3)		
			
N1—C11—C12	122.5 (3)	C37—C36—H36	119.1
N1—C11—H11	118.7	C38—C37—C36	119.8 (3)
C12—C11—H11	118.7	C38—C37—H37	120.1
C13—C12—C11	119.6 (3)	C36—C37—H37	120.1
C13—C12—H12	120.2	O3—C38—C37	124.2 (3)
C11—C12—H12	120.2	O3—C38—C39	117.1 (2)
N002—C003—H00A	109.5	C37—C38—C39	118.7 (3)
N002—C003—H00B	109.5	N3—C39—C34	123.2 (2)
H00A—C003—H00B	109.5	N3—C39—C38	115.4 (2)
N002—C003—H00C	109.5	C34—C39—C38	121.4 (3)
H00A—C003—H00C	109.5	N4—C41—C42	121.9 (3)
H00B—C003—H00C	109.5	N4—C41—H41	119.1
C12—C13—C14	119.8 (3)	C42—C41—H41	119.1
C12—C13—H13	120.1	C43—C42—C41	119.9 (3)
C14—C13—H13	120.1	C43—C42—H42	120
O001—C004—N002	123.7 (4)	C41—C42—H42	120
O001—C004—H004	118.2	C42—C43—C44	120.1 (3)
N002—C004—H004	118.2	C42—C43—H43	120
C13—C14—C19	116.6 (3)	C44—C43—H43	120
C13—C14—C15	124.9 (3)	C45—C44—C49	118.6 (3)
C19—C14—C15	118.5 (3)	C45—C44—C43	125.1 (3)
C16—C15—C14	119.7 (3)	C49—C44—C43	116.2 (3)
C16—C15—H15	120.2	C46—C45—C44	119.4 (3)
C14—C15—H15	120.2	C46—C45—H45	120.3
N002—C005—H00D	109.5	C44—C45—H45	120.3
N002—C005—H00E	109.5	C45—C46—C47	122.0 (3)
H00D—C005—H00E	109.5	C45—C46—H46	119
N002—C005—H00F	109.5	C47—C46—H46	119
H00D—C005—H00F	109.5	C48—C47—C46	120.5 (3)
H00E—C005—H00F	109.5	C48—C47—H47	119.7
C15—C16—C17	121.6 (3)	C46—C47—H47	119.7
C15—C16—H16	119.2	O4—C48—C47	125.3 (3)
C17—C16—H16	119.2	O4—C48—C49	116.9 (2)
C18—C17—C16	120.8 (3)	C47—C48—C49	117.8 (3)
C18—C17—H17	119.6	N4—C49—C44	123.1 (3)
C16—C17—H17	119.6	N4—C49—C48	115.2 (2)
O1—C18—C17	124.5 (2)	C44—C49—C48	121.6 (3)
O1—C18—C19	117.8 (2)	C11—N1—C19	118.5 (2)
C17—C18—C19	117.7 (2)	C11—N1—Hf1	129.25 (18)
N1—C19—C14	123.0 (2)	C19—N1—Hf1	112.21 (16)
N1—C19—C18	115.2 (2)	C21—N2—C29	118.2 (2)
C14—C19—C18	121.8 (2)	C21—N2—Hf1	128.67 (19)
N2—C21—C22	123.0 (3)	C29—N2—Hf1	113.03 (17)
N2—C21—H21	118.5	C004—N002—C003	122.1 (4)
C22—C21—H21	118.5	C004—N002—C005	119.1 (3)
C23—C22—C21	119.1 (3)	C003—N002—C005	118.8 (3)
C23—C22—H22	120.4	C31—N3—C39	117.9 (2)
C21—C22—H22	120.4	C31—N3—Hf1	129.96 (19)
C22—C23—C24	120.2 (3)	C39—N3—Hf1	112.11 (17)
C22—C23—H23	119.9	C41—N4—C49	118.8 (2)
C24—C23—H23	119.9	C41—N4—Hf1	128.39 (19)
C23—C24—C29	116.5 (3)	C49—N4—Hf1	112.80 (17)
C23—C24—C25	125.3 (3)	H01—O01—H02	97 (2)
C29—C24—C25	118.2 (3)	C18—O1—Hf1	123.31 (16)
C26—C25—C24	119.8 (3)	C28—O2—Hf1	122.97 (17)
C26—C25—H25	120.1	C38—O3—Hf1	123.71 (17)
C24—C25—H25	120.1	C48—O4—Hf1	124.29 (17)
C25—C26—C27	121.9 (3)	O3—Hf1—O4	142.37 (7)
C25—C26—H26	119	O3—Hf1—O1	91.75 (7)
C27—C26—H26	119	O4—Hf1—O1	99.61 (8)
C28—C27—C26	120.4 (3)	O3—Hf1—O2	100.40 (8)
C28—C27—H27	119.8	O4—Hf1—O2	92.65 (8)
C26—C27—H27	119.8	O1—Hf1—O2	141.60 (7)
O2—C28—C27	124.9 (3)	O3—Hf1—N3	71.30 (8)
O2—C28—C29	117.1 (2)	O4—Hf1—N3	78.35 (8)
C27—C28—C29	118.0 (3)	O1—Hf1—N3	142.80 (7)
N2—C29—C24	123.0 (3)	O2—Hf1—N3	75.26 (8)
N2—C29—C28	115.3 (2)	O3—Hf1—N2	73.34 (8)
C24—C29—C28	121.7 (3)	O4—Hf1—N2	144.02 (7)
N3—C31—C32	122.6 (3)	O1—Hf1—N2	78.33 (8)
N3—C31—H31	118.7	O2—Hf1—N2	70.75 (8)
C32—C31—H31	118.7	N3—Hf1—N2	124.52 (8)
C33—C32—C31	119.8 (3)	O3—Hf1—N4	78.49 (8)
C33—C32—H32	120.1	O4—Hf1—N4	70.70 (8)
C31—C32—H32	120.1	O1—Hf1—N4	73.72 (7)
C32—C33—C34	119.8 (3)	O2—Hf1—N4	144.28 (7)
C32—C33—H33	120.1	N3—Hf1—N4	70.60 (8)
C34—C33—H33	120.1	N2—Hf1—N4	139.28 (8)
C33—C34—C35	125.2 (3)	O3—Hf1—N1	141.74 (7)
C33—C34—C39	116.6 (3)	O4—Hf1—N1	75.47 (7)
C35—C34—C39	118.2 (3)	O1—Hf1—N1	70.91 (7)
C36—C35—C34	120.0 (3)	O2—Hf1—N1	77.35 (8)
C36—C35—H35	120	N3—Hf1—N1	140.80 (8)
C34—C35—H35	120	N2—Hf1—N1	69.91 (8)
C35—C36—C37	121.8 (3)	N4—Hf1—N1	125.15 (8)
C35—C36—H36	119.1		
			
N1—C11—C12—C13	2.0 (4)	C48—C49—N4—C41	−178.1 (2)
C11—C12—C13—C14	−1.5 (4)	C44—C49—N4—Hf1	179.2 (2)
C12—C13—C14—C19	0.6 (4)	C48—C49—N4—Hf1	1.4 (3)
C12—C13—C14—C15	179.5 (3)	C17—C18—O1—Hf1	173.9 (2)
C13—C14—C15—C16	−180.0 (3)	C19—C18—O1—Hf1	−7.1 (3)
C19—C14—C15—C16	−1.1 (4)	C27—C28—O2—Hf1	170.7 (2)
C14—C15—C16—C17	1.5 (4)	C29—C28—O2—Hf1	−8.7 (3)
C15—C16—C17—C18	−0.2 (4)	C37—C38—O3—Hf1	175.6 (2)
C16—C17—C18—O1	177.6 (2)	C39—C38—O3—Hf1	−5.6 (3)
C16—C17—C18—C19	−1.5 (4)	C47—C48—O4—Hf1	178.6 (2)
C13—C14—C19—N1	−0.2 (4)	C49—C48—O4—Hf1	−3.0 (3)
C15—C14—C19—N1	−179.2 (2)	C38—O3—Hf1—O4	−32.6 (2)
C13—C14—C19—C18	178.4 (2)	C38—O3—Hf1—O1	−140.84 (19)
C15—C14—C19—C18	−0.6 (4)	C38—O3—Hf1—O2	75.8 (2)
O1—C18—C19—N1	1.4 (3)	C38—O3—Hf1—N3	5.36 (18)
C17—C18—C19—N1	−179.5 (2)	C38—O3—Hf1—N2	141.9 (2)
O1—C18—C19—C14	−177.3 (2)	C38—O3—Hf1—N4	−67.84 (19)
C17—C18—C19—C14	1.8 (4)	C38—O3—Hf1—N1	158.48 (18)
N2—C21—C22—C23	0.4 (5)	C48—O4—Hf1—O3	−34.1 (3)
C21—C22—C23—C24	1.8 (5)	C48—O4—Hf1—O1	71.6 (2)
C22—C23—C24—C29	−1.7 (5)	C48—O4—Hf1—O2	−144.9 (2)
C22—C23—C24—C25	179.8 (3)	C48—O4—Hf1—N3	−70.6 (2)
C23—C24—C25—C26	178.5 (3)	C48—O4—Hf1—N2	154.93 (18)
C29—C24—C25—C26	0.0 (5)	C48—O4—Hf1—N4	2.73 (19)
C24—C25—C26—C27	0.1 (5)	C48—O4—Hf1—N1	138.9 (2)
C25—C26—C27—C28	0.2 (5)	C18—O1—Hf1—O3	−138.65 (19)
C26—C27—C28—O2	−179.8 (3)	C18—O1—Hf1—O4	77.37 (19)
C26—C27—C28—C29	−0.4 (4)	C18—O1—Hf1—O2	−29.4 (2)
C23—C24—C29—N2	−0.5 (4)	C18—O1—Hf1—N3	160.71 (17)
C25—C24—C29—N2	178.1 (3)	C18—O1—Hf1—N2	−66.06 (19)
C23—C24—C29—C28	−178.8 (3)	C18—O1—Hf1—N4	143.9 (2)
C25—C24—C29—C28	−0.2 (4)	C18—O1—Hf1—N1	6.51 (18)
O2—C28—C29—N2	1.5 (4)	C28—O2—Hf1—O3	76.4 (2)
C27—C28—C29—N2	−178.0 (3)	C28—O2—Hf1—O4	−139.1 (2)
O2—C28—C29—C24	179.9 (3)	C28—O2—Hf1—O1	−30.0 (3)
C27—C28—C29—C24	0.4 (4)	C28—O2—Hf1—N3	143.7 (2)
N3—C31—C32—C33	−0.2 (4)	C28—O2—Hf1—N2	8.28 (19)
C31—C32—C33—C34	−0.1 (4)	C28—O2—Hf1—N4	161.10 (18)
C32—C33—C34—C35	179.7 (3)	C28—O2—Hf1—N1	−64.6 (2)
C32—C33—C34—C39	1.0 (4)	C31—N3—Hf1—O3	176.5 (2)
C33—C34—C35—C36	−177.8 (3)	C39—N3—Hf1—O3	−4.38 (16)
C39—C34—C35—C36	0.8 (4)	C31—N3—Hf1—O4	−26.0 (2)
C34—C35—C36—C37	0.8 (5)	C39—N3—Hf1—O4	153.08 (18)
C35—C36—C37—C38	−1.6 (4)	C31—N3—Hf1—O1	−116.6 (2)
C36—C37—C38—O3	179.6 (3)	C39—N3—Hf1—O1	62.5 (2)
C36—C37—C38—C39	0.7 (4)	C31—N3—Hf1—O2	69.9 (2)
C33—C34—C39—N3	−1.6 (4)	C39—N3—Hf1—O2	−111.01 (18)
C35—C34—C39—N3	179.6 (2)	C31—N3—Hf1—N2	123.4 (2)
C33—C34—C39—C38	177.1 (2)	C39—N3—Hf1—N2	−57.50 (19)
C35—C34—C39—C38	−1.7 (4)	C31—N3—Hf1—N4	−99.4 (2)
O3—C38—C39—N3	0.8 (3)	C39—N3—Hf1—N4	79.64 (18)
C37—C38—C39—N3	179.7 (2)	C31—N3—Hf1—N1	22.8 (3)
O3—C38—C39—C34	−178.0 (2)	C39—N3—Hf1—N1	−158.09 (15)
C37—C38—C39—C34	0.9 (4)	C21—N2—Hf1—O3	70.0 (2)
N4—C41—C42—C43	0.3 (4)	C29—N2—Hf1—O3	−114.43 (19)
C41—C42—C43—C44	−0.2 (4)	C21—N2—Hf1—O4	−115.8 (2)
C42—C43—C44—C45	177.8 (3)	C29—N2—Hf1—O4	59.8 (2)
C42—C43—C44—C49	−0.2 (4)	C21—N2—Hf1—O1	−25.4 (2)
C49—C44—C45—C46	−0.8 (4)	C29—N2—Hf1—O1	150.15 (19)
C43—C44—C45—C46	−178.7 (3)	C21—N2—Hf1—O2	177.7 (3)
C44—C45—C46—C47	0.3 (5)	C29—N2—Hf1—O2	−6.74 (17)
C45—C46—C47—C48	1.2 (5)	C21—N2—Hf1—N3	122.2 (2)
C46—C47—C48—O4	176.5 (3)	C29—N2—Hf1—N3	−62.2 (2)
C46—C47—C48—C49	−1.9 (4)	C21—N2—Hf1—N4	21.8 (3)
C45—C44—C49—N4	−177.7 (2)	C29—N2—Hf1—N4	−162.60 (16)
C43—C44—C49—N4	0.4 (4)	C21—N2—Hf1—N1	−99.2 (2)
C45—C44—C49—C48	0.0 (4)	C29—N2—Hf1—N1	76.41 (18)
C43—C44—C49—C48	178.1 (3)	C41—N4—Hf1—O3	−24.6 (2)
O4—C48—C49—N4	0.6 (3)	C49—N4—Hf1—O3	155.97 (18)
C47—C48—C49—N4	179.2 (2)	C41—N4—Hf1—O4	177.4 (2)
O4—C48—C49—C44	−177.2 (2)	C49—N4—Hf1—O4	−2.10 (17)
C47—C48—C49—C44	1.4 (4)	C41—N4—Hf1—O1	70.7 (2)
C12—C11—N1—C19	−1.6 (4)	C49—N4—Hf1—O1	−108.75 (18)
C12—C11—N1—Hf1	176.48 (18)	C41—N4—Hf1—O2	−116.4 (2)
C14—C19—N1—C11	0.7 (4)	C49—N4—Hf1—O2	64.1 (2)
C18—C19—N1—C11	−178.0 (2)	C41—N4—Hf1—N3	−98.6 (2)
C14—C19—N1—Hf1	−177.7 (2)	C49—N4—Hf1—N3	81.95 (18)
C18—C19—N1—Hf1	3.6 (3)	C41—N4—Hf1—N2	22.2 (3)
C22—C21—N2—C29	−2.6 (4)	C49—N4—Hf1—N2	−157.26 (16)
C22—C21—N2—Hf1	172.8 (2)	C41—N4—Hf1—N1	122.2 (2)
C24—C29—N2—C21	2.7 (4)	C49—N4—Hf1—N1	−57.2 (2)
C28—C29—N2—C21	−178.9 (2)	C11—N1—Hf1—O3	−116.0 (2)
C24—C29—N2—Hf1	−173.4 (2)	C19—N1—Hf1—O3	62.1 (2)
C28—C29—N2—Hf1	5.0 (3)	C11—N1—Hf1—O4	70.9 (2)
O001—C004—N002—C003	178.5 (5)	C19—N1—Hf1—O4	−110.93 (18)
O001—C004—N002—C005	−1.7 (7)	C11—N1—Hf1—O1	176.7 (2)
C32—C31—N3—C39	−0.3 (4)	C19—N1—Hf1—O1	−5.14 (16)
C32—C31—N3—Hf1	178.7 (2)	C11—N1—Hf1—O2	−25.2 (2)
C34—C39—N3—C31	1.3 (4)	C19—N1—Hf1—O2	152.92 (18)
C38—C39—N3—C31	−177.5 (2)	C11—N1—Hf1—N3	21.3 (3)
C34—C39—N3—Hf1	−178.0 (2)	C19—N1—Hf1—N3	−160.53 (16)
C38—C39—N3—Hf1	3.3 (3)	C11—N1—Hf1—N2	−99.1 (2)
C42—C41—N4—C49	−0.1 (4)	C19—N1—Hf1—N2	79.04 (17)
C42—C41—N4—Hf1	−179.53 (19)	C11—N1—Hf1—N4	124.0 (2)
C44—C49—N4—C41	−0.3 (4)	C19—N1—Hf1—N4	−57.80 (19)

### Hydrogen-bond geometry (Å, °)

**Table:**

*D*—H···*A*	*D*—H	H···*A*	*D*···*A*	*D*—H···*A*
O01—H02···O2	0.89 (2)	1.99 (2)	2.867 (3)	171 (3)
O01—H01···O001	0.95 (2)	1.83 (2)	2.757 (4)	164 (4)
C31—H31···O001	0.95	2.51	3.418 (4)	160
C004—H004···O01^i^	0.95	2.41	3.331 (5)	164

Symmetry codes: (i) −*x*, −*y*+1, −*z*.
